# Spatio-Temporal Variability in Accretion and Erosion of Coastal Foredunes in the Netherlands: Regional Climate and Local Topography

**DOI:** 10.1371/journal.pone.0091115

**Published:** 2014-03-06

**Authors:** Joep G. S. Keijsers, Ate Poortinga, Michel J. P. M. Riksen, Jerry Maroulis

**Affiliations:** 1 Soil Physics and Land Management Group, Wageningen University, Wageningen, The Netherlands; 2 School of Science, Education and Engineering, University of the Sunshine Coast, Sippy Downs, Queensland, Australia; University of Aveiro, Portugal

## Abstract

Depending on the amount of aeolian sediment input and dune erosion, dune size and morphology change over time. Since coastal foredunes play an important role in the Dutch coastal defence, it is important to have good insight in the main factors that control these changes. In this paper the temporal variations in foredune erosion and accretion were studied in relation to proxies for aeolian transport potential and storminess using yearly elevation measurements from 1965 to 2012 for six sections of the Dutch coast. Longshore differences in the relative impacts of erosion and accretion were examined in relation to local beach width. The results show that temporal variability in foredune accretion and erosion is highest in narrow beach sections. Here, dune erosion alternates with accretion, with variability displaying strong correlations with yearly values of storminess (maximum sea levels). In wider beach sections, dune erosion is less frequent, with lower temporal variability and stronger correlations with time series of transport potential. In erosion dominated years, eroded volumes decrease from narrow to wider beaches. When accretion dominates, dune-volume changes are relatively constant alongshore. Dune erosion is therefore suggested to control spatial variability in dune-volume changes. On a scale of decades, the volume of foredunes tends to increase more on wider beaches. However, where widths exceed 200 to 300 m, this trend is no longer observed.

## Introduction

Coastal foredunes are an important part of the Dutch coastal landscape since they form a natural flood defence. Foredunes are part of the beach-dune system within which sediment is transferred by aeolian and marine processes. Aeolian sediment transport from the beach contributes to the dune volume, whereas marine processes associated with storm surges erode dune sediments thereby lowering the dune volume. Depending on the balance between erosion and accretion, dune volume and morphology change over time. The ability to model and predict such changes is still limited [1], [2]. This study examines how yearly fluctuations in regional climatic variables contribute to changes in foredune volume and how the balance between these forces is influenced by beach width.

Depending on the spatio-temporal scale of investigation, different environmental variables influence sediment transfers to and from coastal dunes [3]. This paper is focused on meso-scale dune development, which, is controlled by aeolian transport potential and storm intensity [1].

Aeolian transport provides the primary mechanism for sediment input to the dunes. This occurs when wind velocity exceeds the sediment entrainment threshold resulting in sediment being eroded from the beach and transported downwind. The potential for aeolian transport into the dunes for a certain period can be estimated from regional wind data [4]–[6]. Whether the measured sediment input meets the potential depends on the presence of supply-limiting factors, such as surface moisture [7]–[9], crust formation [10], lag deposits [11] and beach width. Beach width determines the maximum fetch, which is the distance downwind where transport takes place. A minimum distance is required for transport to reach a maximum, called the critical fetch distance [12], [13]. If beach width is insufficient for maximum transport to develop, aeolian transport is reduced relative to the transport potential [7], [14], [15]. Aeolian transport is more prevalent on wide beaches, where there is a large supply of sediment for aeolian transport and unrestricted fetch length. Although the highest transport rates are expected during the highest wind velocities, such wind velocities are often accompanied by storm surges and wave run up that reduce the fetch length and increase moisture content of the beach surface and may even erode the dune. Consequently, Delgado-Fernandez and Davidson-Arnott [16] concluded that most of the sediment input to the dunes actually occurs during low- to medium-magnitude wind events.

Detailed studies of coastal foredune erosion provide a comprehensive understanding of the relevant coastal processes and interactions, resulting in the effects of storm events on dune dynamics being accurately predicted. Foredune erosion, which operates at a scale of hours to days, occurs when elevated sea level and wave run-up reach and undermine the dune foot. Storm intensity depends on the meteorological conditions that determine surge level, wave conditions and storm duration [17]–[19]. The volume of sediment that is eroded from the foredune also depends on the angle of wave incidence and on the amount of energy dissipated traversing over sand waves, sand bars and the beach [20], [21]. Therefore, the spatial variability in dune erosion under equivalent storm conditions can be related to differences in coastline orientation [22], longshore variations in inner-shelf geology and sand bars [23], [24], or variations in beach morphology and beach width [25]–[28]. Most eroded sediment resettles on the foreshore [17] and foredunes may recover rapidly if the sediment-transport potential and re-vegetation are sufficient [29].

A critical factor in foredune development is sediment supply from the shoreface to the beach (e.g. [30]–[32]). This sediment supply depends on the welding of nearshore bars (e.g. [30], [31]), gradients in longshore transport [33], [34] or other nearshore processes (e.g. [35]). At timescales of decades to centuries, the relative importance of sediment supply over transport potential increases [1]. However, the factors controlling sediment supply to the beach were not within the focus of this study. Instead, beach width was used to provide an indirect measure of sediment availability for dune building.

Temporal variability in dune volume results from fluctuations in yearly erosion and accretion. The effects of regional climate on dune volume display correlations between storminess and dune erosion [6], [36]; however, there is little evidence linking yearly wind climate and aeolian sediment input to the dunes. Assuming a homogenous wind and longshore wave climate, spatial variability in dune volume is likely to be related to local beach morphology. A number of recent studies investigated foredunes in relation to beach morphology and found that foredune accretion was dominant when beaches were wider than a site-specific critical width [37], when beach slopes were relatively gentle [6], or where sand banks were welded to the shoreline [38]. Further identification and testing of meso-scale controls on foredune development are needed to improve predictions and modelling of environmental-change impacts and management interventions on coastal dunes.

This study investigates how the balance between erosion and accretion is controlled by regional climate and local morphology. On the basis of yearly dune volumes, hourly sea levels and wind data, we investigate (1) the temporal variability in erosion and accretion in relation to variations in storminess and aeolian transport potential; (2) the influence of beach width on dune erosion and accretion; and (3) the decadal effect of beach width on dune development.

## Methods

### Regional Setting

**Figure 1:**
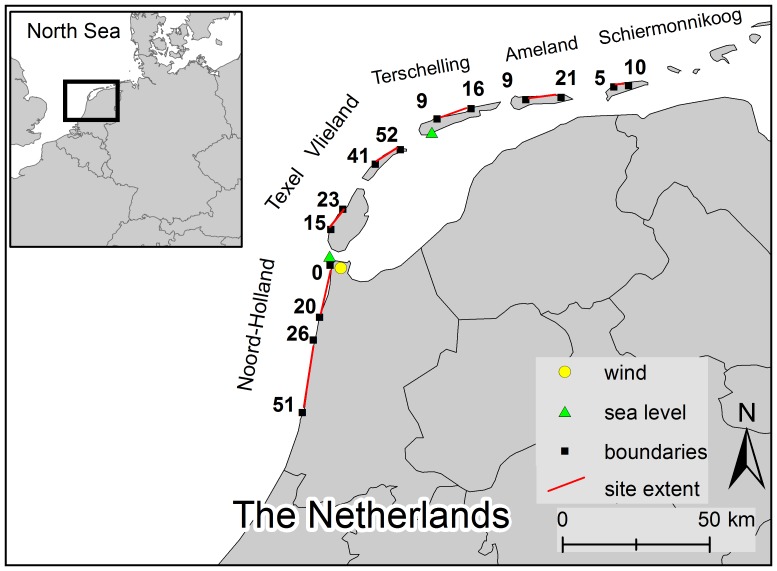
Study areas. Map shows the location of the six coastal sections used in this study within the Netherlands and identifies the location of the wind gauge (KNMI station De Kooy) and two sea-level gauges (Den Helder and West Terschelling). Positions and numbers of beach poles on the section boundaries are indicated. Inset shows the location of the study area within Northwestern Europe.

Six sections of the Dutch coastline were selected for analysis. In a convex line from west to east, these are Noord-Holland, Texel, Vlieland, Terschelling, Ameland and Schiermonnikoog (Fig. 1). The sections are separated by tidal inlets, connecting the North Sea to the Wadden Sea. Except for Noord-Holland, all locations are barrier islands, and together, they cover 195 km of the Dutch coast (Fig. 1). Prevailing winds are from the south-west. The tidal range varies between 1.6 m in Noord-Holland and 2.1 m in Schiermonnikoog. Mean grain size of natural beach sediment is 259 µm in Noord-Holland and decreases to 202 µm on Ameland [39] and 190 µm on Schiermonnikoog [40].

Compared to the other sites, beaches of Noord-Holland and Texel are narrow (<100 m) and show limited temporal variability. The other barrier islands feature wider beaches (>100 m) with larger spatio-temporal variations, influenced by morphodynamics of tidal inlets (e.g. [41], [42]). Widest beaches are found on the updrift (western) heads of the islands.

All sites are characterised by sandy beaches, backed by a continuous foredune ridge that is partly covered by marram grass (*Ammophila arenaria*). Average dune height ranges between 16 m +NAP (Noord-Holland) and 8 m +NAP (Schiermonnikoog), where NAP is the Dutch vertical datum approximating mean sea level. The majority of the foredunes has been influenced by vegetation plantings, sand fences or sand nourishments (Arens, 1994). Natural foredunes are found at the extremities of the islands, where beaches with mobile dune fields are present [42], [43].

Vegetation plantings and sand fences enhance sedimentation, but do not strongly interfere with natural foredune-development processes [44]. Sand nourishment, however, can change the sediment budget of the beach and foredune, especially when nourishments are applied directly to the beach and dune, which changes the volume of available sediment and the morphology. Since 1990, the Dutch coastal policy [45] ensures that sand nourishments are placed on the shoreface and the beach, thereby reducing any direct impact on the foredune; a process that may still influence dune development by protecting the dunes against erosion, and by changing the sediment source characteristics for aeolian sand transport [39], [46].

### Data Collection and Preparation

Cross-shore elevation profiles over the period 1965 to 2012 were obtained from the JARKUS dataset. This dataset contains annual elevation measurements covering the dune, beach and foreshore and has been used in several studies addressing annual- to decadal-scale behaviour of the coastline [6], [39], [47], [48].

Profiles are spaced 200 to 250 m apart, coinciding with beach poles along the Dutch coast. Elevation measurements along the transects were taken at 5 m intervals [39]. Until 1977, the sub-aerial beach was measured by levelling, then aerial photography was used from 1978 to 1995, and since then, laser altimetry [47]. The reported measurement errors (σ) of the techniques differ substantially, from 0.01 m for levelling [49], to 0.1 m for photogrammetry [50] and laser altimetry [51], [52].

The longshore extent of sections in this study is constrained by the limits of a homogenous coastline orientation. Consequently, the protruding seawall (‘Hondsbossche Zeewering’) near Petten was omitted, which explains the gap between profiles 20 and 26 for Noord-Holland (Table 1).

**Table 1:** Longshore extent of the six coastal sections, showing the total number of profile measurements available for the section and the number of profiles discarded.

Name	Alongshore extent (km)	n observations	n discarded
Noord-Holland	0–19.9, 26–51	7564	723
Texel	15–23	1276	152
Vlieland	42–52	1743	55
Terschelling	9–16	983	12
Ameland	9–21	1667	176
Schiermonnikoog	5–10	955	92
Total:	89	14228	1210 (9%)

**Figure 2:**
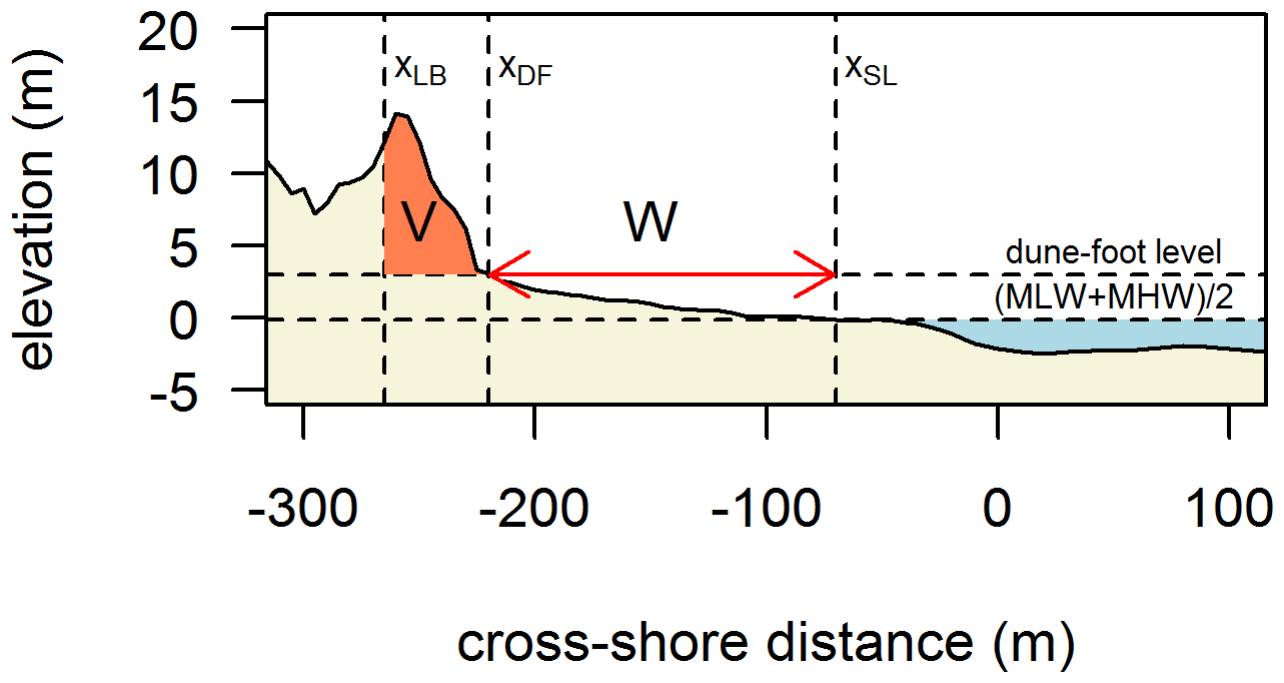
Definition of dune volume and beach width. Position of landward boundary (X_LB_), dune-foot position (X_DF_) and shoreline position (X_SL_) for the black profile. Dune volume (V) and beach width (W) were calculated on the basis of these positions.

Two parameters were calculated from the yearly elevation profile: sub-aerial beach width (W in m) and dune volume (V in m^3^/m). Beach width is defined as the distance between the shoreline (X_SL_) and dune-foot (X_DF_), while dune volume is the volume of sediment per m longshore above the dune-foot level, seaward of a fixed inland boundary (X_LB_). X_DF_ is the most seaward position where dune-foot level is reached. This level is taken as 3 m+NAP, which is the elevation at which the profile slope changes significantly [39], [47]. X_LB_ is the farthest-inland crest position in a profile’s time series and the shoreline (X_SL_) is the cross-shore position where elevation is equal to the mean of the average low- (MLW) and high-tide (MHW) positions [27], [53] (Figure 2). Finally, the difference between two consecutive values of V yields the change in dune volume ΔV, which represents the parameter of interest in this study.

Two filters were used to identify and eliminate outliers in calculations of dune-volume change (ΔV) that are caused by human activities and measurement errors. Firstly, the *nourishment filter* discards values of ΔV directly following sand nourishment. This filter includes all profiles in the zone of the sand nourishment and a buffer zone of 300 m on either side. This discards the profiles directly bordering the nourished zone, as these were found to show considerable modifications in beach morphology following the nourishment. Such modifications were not observed in profiles further away. Secondly, the *dune-foot residuals filter* discards any profile measurement that displays a sudden dune-foot movement >50 m. This distance lies 3 standard deviations from the mean and movements >50 m are therefore considered outliers, caused by measurement errors or by the formation of a short-lived incipient dune, seaward of the actual foredune. Of the 14228 available profile measurements, 1210 or 9% were discarded after these two filters were applied (Table 1).

### Storminess

Storminess is a complex set of environmental conditions that may lead to dune erosion, such as powerful onshore wind, high-energy waves and high water levels. Several parameters have been defined and tested to quantify storminess on a yearly timescale (e.g. [48]). Assuming that the erosion impact of a storm is determined by the highest recorded water level, then the yearly storminess (S) is defined as the maximum level recorded between two profile measurements [27]. This parameter was found to explain some of the year-to-year variability in dune-foot movement [27] and dune volume [6]. Yearly values of S are derived from hourly sea levels, which are measured at a number of tide stations along the coast, of which Den Helder and West Terschelling are within the study area (Figure 1). Given that both the correlation between these tide stations is high (r = 0.93) and that data at West Terschelling are available from 1965 to 2012, the record from this latter station was used for all sites. Correlation between storminess and dune-volume changes was calculated using the Pearson product-momentum. The Pearson r takes a value between −1 and +1, where −1 indicates perfect negative correlation and 1 perfect positive correlation. The significance of the correlation was tested at the p<0.05 level.

### Transport Potential

Transport potential is an indicator for the potential aeolian transport into the dunes based on wind velocity and wind direction. Transport potential can be calculated by applying a time series of regional wind data to aeolian transport equations (e.g. [54]–[57]). Transport potential is related to the cube of shear velocity; therefore, high shear velocities associated with storm winds dominate the final value for transport potential. However, this does not agree with the notion that low- to medium-magnitude winds are most important for actual aeolian input into the dunes [16]. Therefore, two time series of transport potential were calculated. The first series uses the full range of measured wind velocities (Q_all_). The second series uses only wind velocities below a given value. As there were no local field measurement, wind velocities of 8 m/s (Q_8_), 10 m/s (Q_10_) and 12 m/s (Q_12_) were tested as the upper limit for aeolian transport potential.

The yearly transport potential (Q) was calculated as a measure of aeolian forcing [54], [57]. Hourly values of wind velocity at 10 m above the surface were measured and provided by the Royal Dutch Meteorological Institute (KNMI). Hourly values of wind direction and velocity from the KNMI station De Kooy (Fig. 1) were converted to shear-velocity values using the law of the wall:
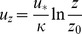
(1)where u_z_ is the wind speed (m/s) at elevation *z* above the bed (m), u_*_ is the shear velocity (m/s), *κ* the von Kármán constant (0.4) and *z_0_* the roughness length, taken as 0.001 m [11].

The threshold shear velocity for transport is then calculated as
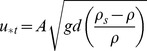
(2)where u_*t_ is the threshold shear velocity (m/s), A is a dimensionless constant (0.1 for the impact threshold), *g* is the gravitational acceleration (m/s^2^), *d* is median grain size in the field, ρ_s_ is the density of the sediment (kg/m^3^) and ρ is the density of air (kg/m^3^). As differences in grain sizes were relatively small, a median grain size of 0.25 mm was used for all sections.

Hourly potential transport *q_j_* (kg/m/h) was computed whenever hourly u_*_>u_*t_ using the Bagnold equation [58]:
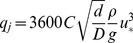
(3)where *C* is a dimensionless empirical constant (1.8), and *D* the grain diameter of a standard sand (0.25 mm).

Fluxes were summed over all directions *i* (10° bins) and wind velocities *j* (0.1 m/s bins) to yield the total amount of sediment that potentially crosses the dune foot in one year (Q):
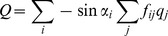
(4)where α*_i_* is the angle of incidence of the wind, *f_ij_* frequency of wind direction *i* and wind velocity *j* (hours) and *q_j_* is the potential aeolian transport for velocity *j*.

Lastly, potential aeolian transport into the dunes was converted from kg/m to m^3^/m (bulk density of 1590 kg/m^3^) to ensure values are comparable with calculated dune volumes.

As wind measurements are available from 1981 onwards, correlations between transport potential and dune-volume change only concern data from 1981 to 2012. Correlation between the time series of potential transport and dune-volume changes were calculated as the Pearson product-momentum correlation coefficient. The significance of the Pearson product-momentum correlation coefficient was tested at the p<0.05 level.

The derived time series of dune volume, beach width, transport potential and maximum water level are available at doi: 10.4121/uuid:54ed4c8f-e7b6-4139-bc20-dc8168c2f890.

## Results

### Temporal Variability in Dune-Volume Changes, Erosional and Accretional Forces

**Figure 3:**
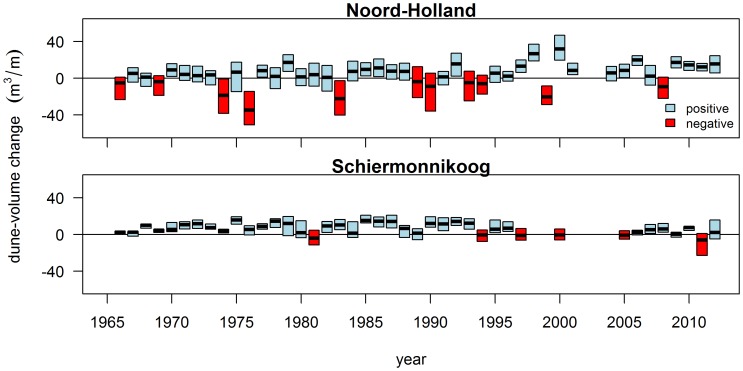
Temporal variability in dune-volume changes. Range of dune-volume changes (ΔV) per year, calculated for Noord-Holland (top) and Schiermonnikoog (bottom). Each boxplot represents the upper and lower quartile and median of dune-volume changes for a single year. Differences among years indicate *temporal* variability in dune-volume changes. The height of the boxes indicates *spatial* variability in dune-volume changes. Boxes with a positive median are in blue, boxes with a negative median are in red.

Dune-volume changes (ΔV) for all sites are generally between −50 and +50 m^3^/m, with average values ranging from −2 at Ameland to +13 m^3^/m at Terschelling. Within any year, there is significant longshore variability in ΔV (Fig. 3). The interquartile distance commonly exceeds 20 m^3^/m and tends to be larger when the median of ΔV values is negative (e.g. 1974, 1976, 1990).

Between years, there are also large differences in ΔV. This temporal (year-to-year) variability in ΔV is apparent from the strongly different median and quartiles of ΔV (Fig. 3). For Noord-Holland, longshore average ΔV ranges from −35 to 31 m^3^/m. The lowest value, for 1976, corresponds to a 1-in-20 years storm [6]. In most years, however, average ΔV is positive, which indicates dune growth. Temporal variability is lowest on Schiermonnikoog (Fig. 3), Vlieland and Terschelling (not shown).

**Figure 4:**
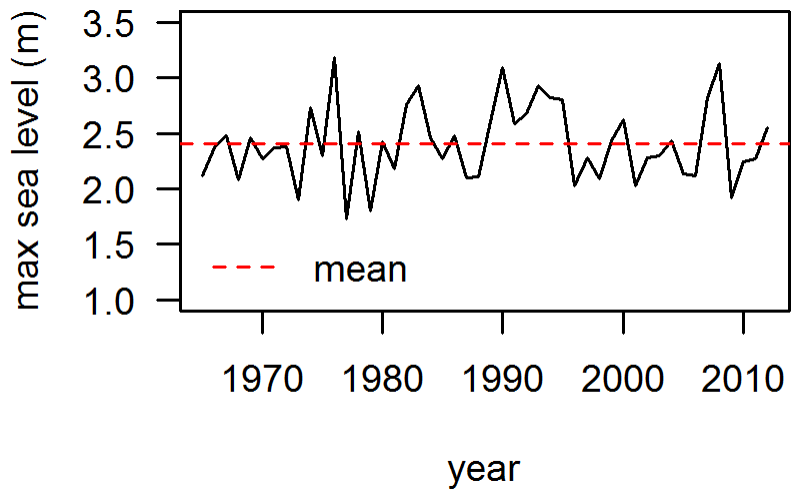
Yearly maximum sea level. Yearly maximum sea level as measured at the tide station of West-Terschelling (1965–2012).

The indicator for storminess (S) shows considerable temporal variation (Fig. 4). The highest sea levels were recorded in 1976, 1990 and 2008 and caused significant dune erosion (e.g. [59]). Values of S<2 m occurred in 1973, 1977, 1979 and 2009, causing minor dune erosion only in 1973 [60]. Note that the years listed here do not refer to calendar years, but to profile-to-profile cycles.

**Figure 5:**
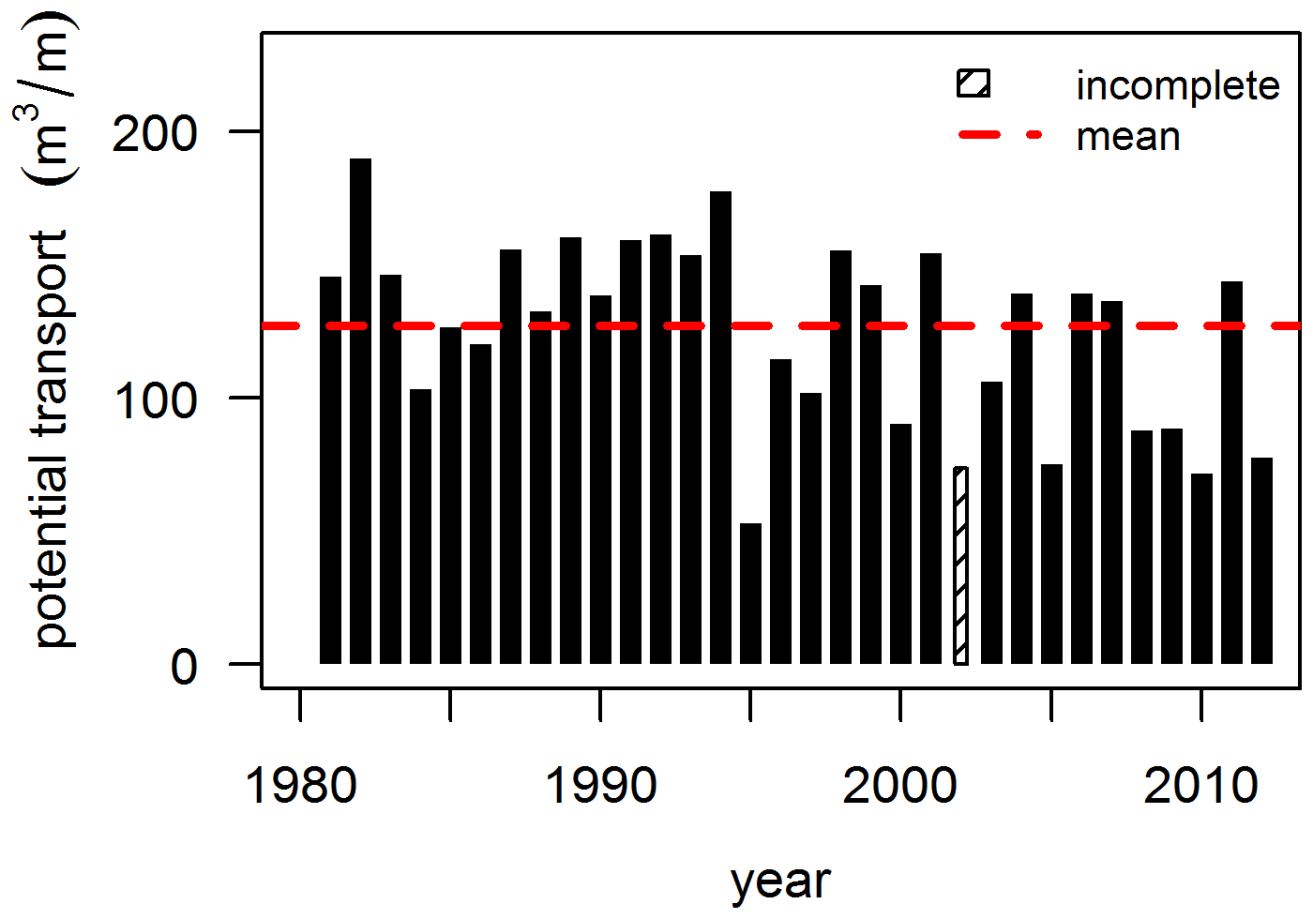
Yearly transport potential. Yearly transport potential for location Noord-Holland. The measurements of 2002 are incomplete, with 22 hours of missing data. This hiatus seems unrelated to storm conditions. The mean transport potential is 125 m^3^/m.

Dune accretion is linked with transport potential (Q), which also shows considerable temporal variability for all sites, caused by the year-to-year variations in wind climate (Fig. 5). The average transport potential is highest in Noord-Holland (125 m^3^/m) and decreases, as the shoreline orientation changes from west to north, to 40 m^3^/m at Ameland and Schiermonnikoog (Table 2). This decrease in transport potential reflects the changing orientation relative to the dominant south-west wind direction. Potential sediment input calculated from only those hours with wind velocities below 8, 10 or 12 m/s (Q_8,_ Q_10,_ Q_12)_ displayed similar variability. However, the mean values were reduced relative to Q_all_ as the latter includes all wind velocities.

**Table 2:** Average and standard deviation of yearly transport potential, calculated from 1980–2012 data.

		transport potential (m^3^/m)	
Location	Shoreline orientation (°)	mean	st. dev.
Noord-Holland	190	127	34
Texel	215	83	25
Vlieland	235	61	18
Terschelling	255	45	11
Ameland	265	40	9
Schiermonnikoog	265	40	9

### Influence of Erosional and Accretional Forces on Dune Volume

**Figure 6:**
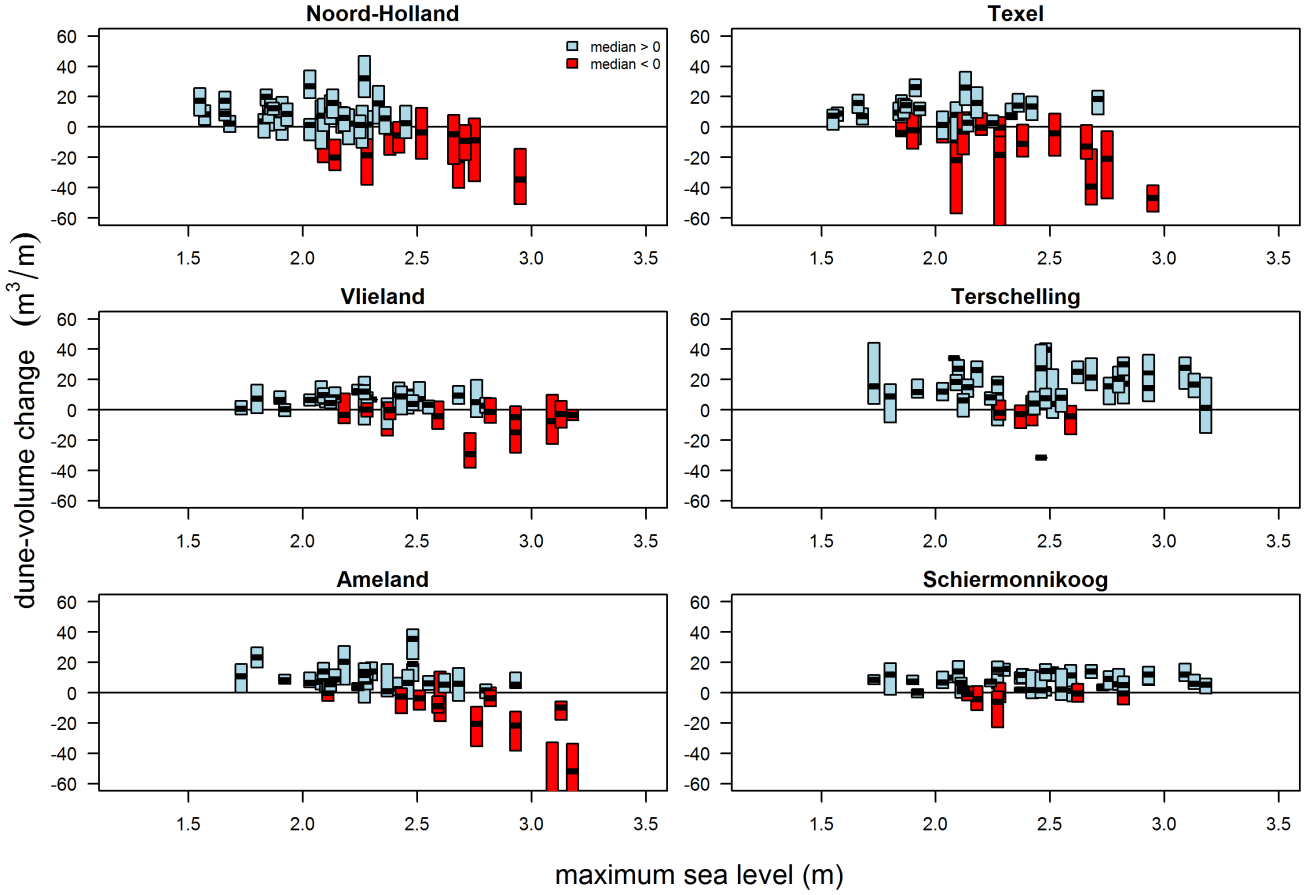
Dune-volume changes (ΔV) as a function of maximum sea level (S) for all sites. Each boxplot represents the longshore variation for a single year. Boxplots with a positive median in blue, with a negative median in red.

Dune erosion (negative ΔV) is linked with high values of S (Fig. 6). For Noord-Holland, ΔV is mainly negative when S>2.5 m, which indicates that the eroded sediment volume is larger than the accreted volume. When S is between 2.0 and 2.5 m, ΔV can be both positive and negative. When S<2.0 m, positive values for ΔV dominate (Fig. 6). Similar links exist between S and longshore-averaged ΔV for Texel, Vlieland and Ameland. Both Terschelling and Schiermonnikoog show a lower occurrence of negative ΔV and no obvious relationship between S and ΔV (Fig. 6).

**Figure 7:**
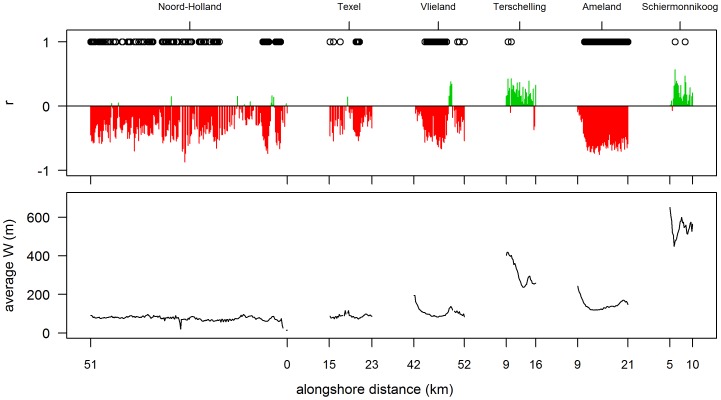
Temporal correlation of dune-volume changes (ΔV) and storminess (S). Upper panel: correlation between dune-volume changes (ΔV) and storminess (S). Correlation was calculated as the Pearson product-moment coefficient r. Correlations significant at p<0.05 level are indicated with ‘o’. Lower panel: time-averaged beach width (W) for each profile. The numbers on the x-axis refer to the boundaries of each location.

Time series of both ΔV and S were correlated in Figure 7, showing the strongest correlations for locations where beaches were narrow; with 38% of the correlations being significant. Negative correlations imply that higher values of S are associated with lower values of ΔV. In contrast, correlations are weakly positive on the wide beaches of Terschelling and Schiermonnikoog.

**Figure 8:**
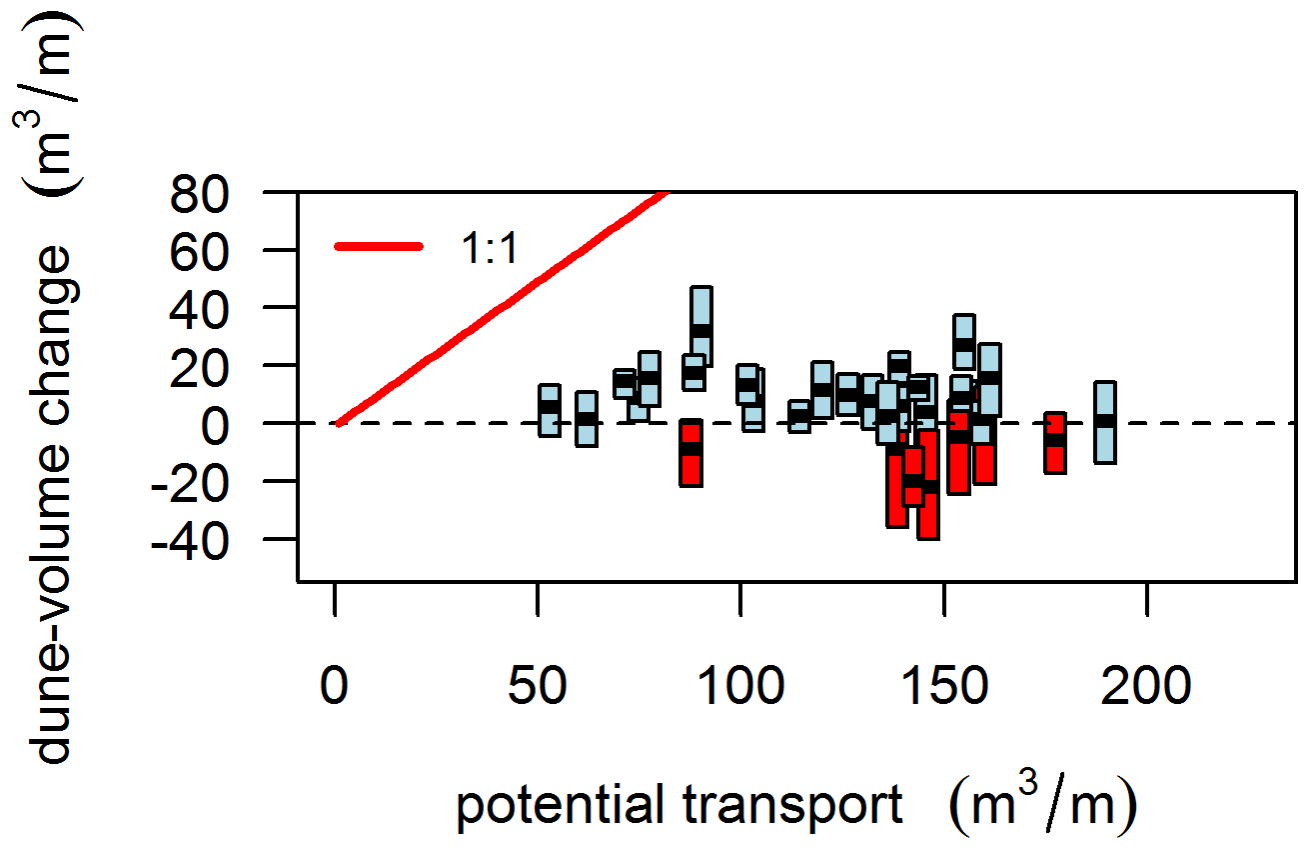
Dune-volume changes (ΔV) as function of transport potential for Noord-Holland. Each boxplot represents the alongshore variation of a single year. Boxplots with a positive median in blue, boxplots with a negative median in red. The red line indicates the 1∶1 line, where potential transport equals ΔV.

Dune accretion is not linked with transport potential (Fig. 8). Values of ΔV are generally below the potential sediment input (Fig. 8), indicating an overestimation of Q relative to the actual volume gain.

Time series of ΔV show a weak correlation with yearly values of Q. For most of the longshore positions, the correlation coefficient is negative (9% were significant), suggesting that increasing Q is associated with decreasing ΔV. Positive correlations are associated primarily with wider beaches, e.g. positive correlations were evident for parts of Vlieland and Ameland and for the islands of Terschelling and Schiermonnikoog. These are, however, very weak (r <0.4) and in only 5% of the cases, a positive correlation is significant.

**Figure 9:**
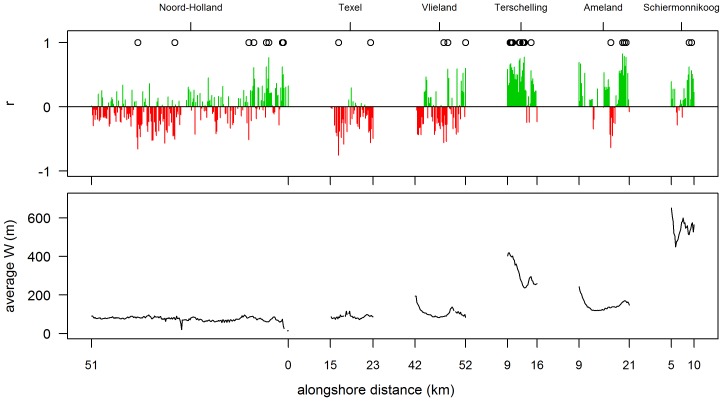
Correlation between time-series of dune-volume changes (ΔV) and transport potential (Q). Upper panel: correlation between ΔV and Q, after discarding years with S>2.5 m. Correlation was calculated as the Pearson product-moment coefficient r. Correlations significant at p<0.05 level are indicated with ‘o’. Lower panel: time-averaged beach width (W) for each profile. The numbers on the x-axis refer to the boundaries of each location.

The low number of significant correlations between Q and ΔV is most probably caused by two different effects. Firstly, strong winds associated with storm surges were included in the analysis. Secondly, within a given year, both dune erosion and dune accretion can occur. Even if aeolian transport is high, a single dune-erosion event may offset or undo any dune accretion. To limit the effect of co-occurring dune accretion and erosion, correlations were re-tested after discarding the years in which S>2.5 m (13 years discarded, 20 remaining). This is the value of S above which erosion dominates accretion (Fig. 6). Discarding these years significantly improved the results, especially for Terschelling, Ameland and Schiermonnikoog (Fig. 9); the results did not differ significantly between Q, Q_8_, Q_10_ and Q_12._ However, compared to the correlations between storminess and ΔV, the explanatory value of Q is still low, with only 5% of the profiles having a significant positive correlation.

### Influence of Beach Width on Dune-Volume Changes

Longshore variations in correlations between the climatic variables and ΔV indicate longshore differences in the balance between erosion and accretion. To investigate how longshore variations are related to beach width, ΔV were correlated with beach width for: (1) erosion-dominated years and (2) accretion-dominated years.

**Figure 10:**
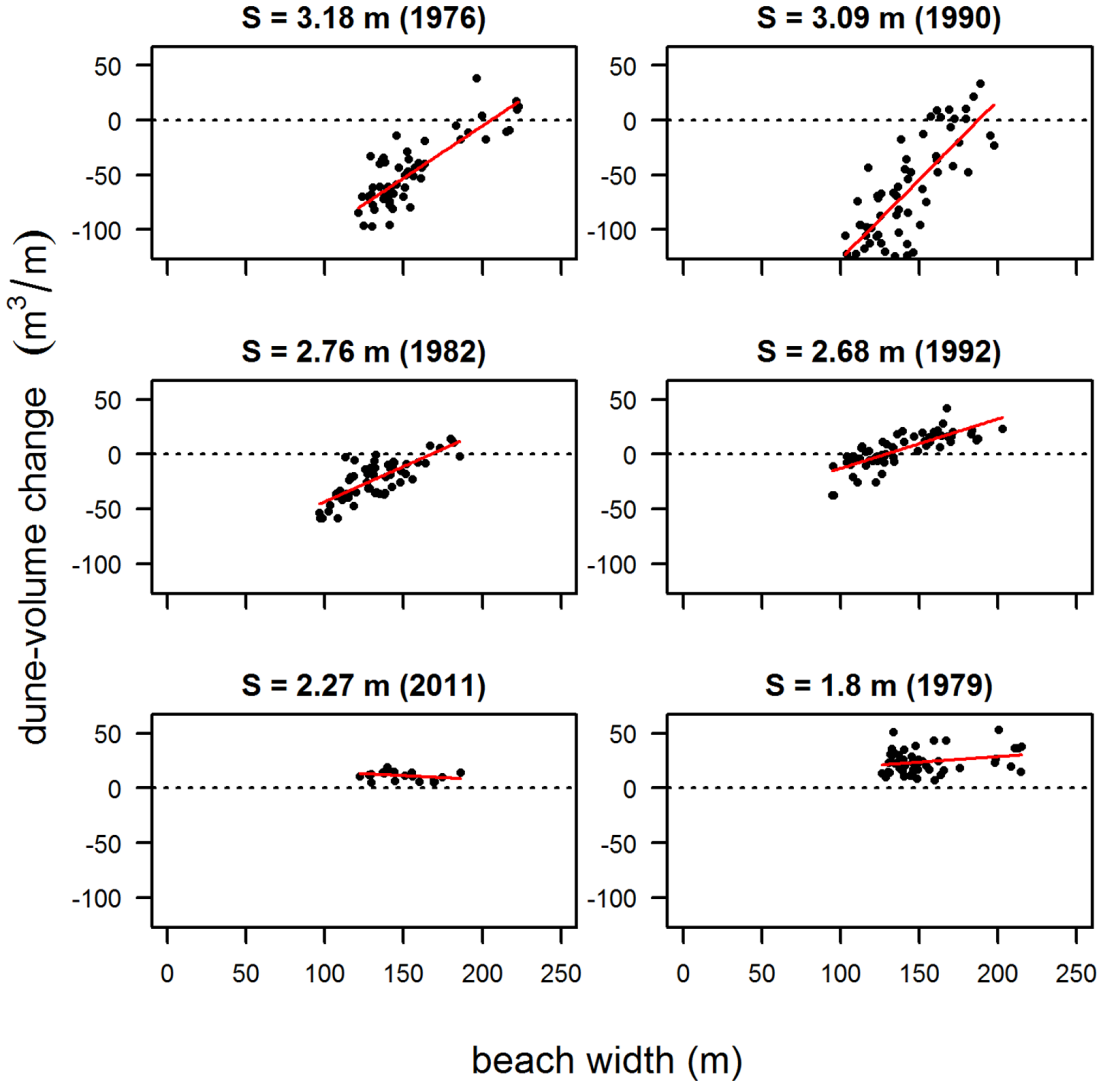
Correlation between dune-volume change (ΔV) and beach width (W) for different values of storminess (S). Data from section Ameland. The red line represents the least squares linear regression.

In years with the highest storminess (S>3 m), although there is a large amount of scatter, a positive correlation can be identified between ΔV and W for Noord-Holland, Texel, Ameland and Vlieland, indicating that wider beaches experience less erosion than narrow beaches (Figure 10, upper panels). This positive trend was observed for W between 50 and 200 m. Where beaches are wider (W>200 m, e.g. Terschelling, Schiermonnikoog), the slope of the correlation is close to 0.

As S decreases from 3 to 2.5 m, the slope of the correlation between ΔV and W becomes less steep (Fig. 10, middle panels). When S<2.5 m (relatively low storminess), the slope approaches 0 (Fig. 10, lower panels), indicating that in accretion-dominated years, values of ΔV are similar across all beach widths.

**Figure 11:**
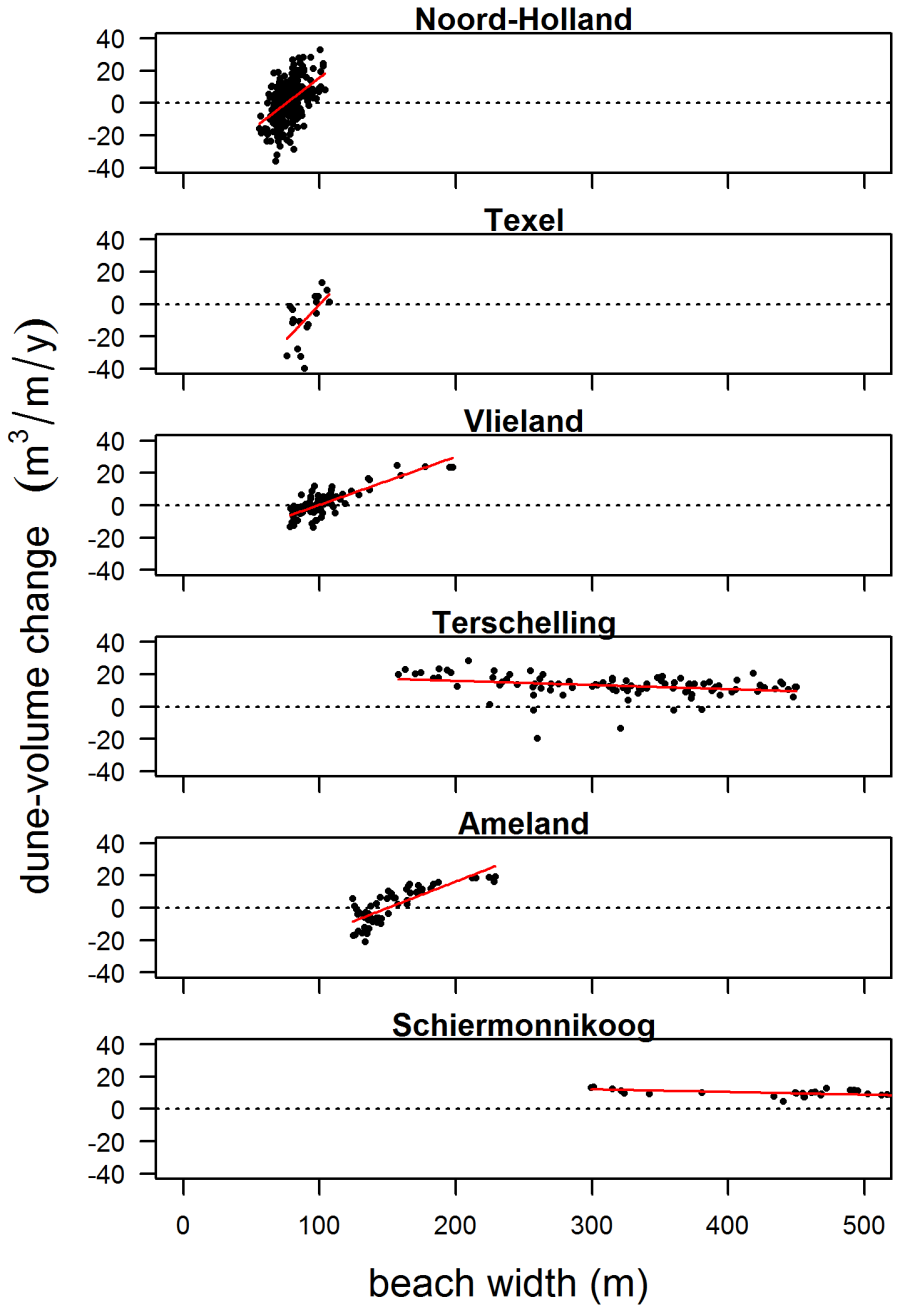
Decadal dune-volume changes (ΔV) as a function of 10-y-averaged beach width (W). Red line indicates least-squares linear fit. All correlations are significant at the p<0.05 level. Values representing a profile with nourishment activity within the specific decade were discarded.

Dune-volume changes on a decadal scale (i.e. differences between dune volume in 1970 and 1980, 1980 and 1990 etc.) integrate the effects of yearly dune accretion and dune erosion. Hence, this scale provides an indication of the relative contributions of dune accretion and dune erosion. At this scale, ΔV is correlated with W (Fig. 11). Although there is a large amount of scatter, a significant positive correlation can be identified for sites with W<200 m, implying that increasing ΔV is associated with increasing W. At Terschelling and Schiermonnikoog, where W>200 m, correlations are slightly negative although values of ΔV are mainly positive, which implies that the amount of accretion is generally larger than the amount of erosion regardless of beach width. Negative ΔV occurs where W <150 m. This indicates that at these sites with narrow beaches, erosion may dominate accretion.

## Discussion

### Temporal Variability in Erosion and Accretion

Temporal variability in ΔV is best explained by the variation in erosive forces rather than aeolian transport potential, as identified by De Vries et al. (2012) [6]. However, the results presented here show that relationships between climatic variables and ΔV fluctuate longshore.

Significant negative correlations between storminess and ΔV were found on beaches <200 m in width. Hence, the temporal variability in ΔV on the associated profiles is dominated by variations in storminess. On wider beaches, no significant correlation was found.

Correlations between time series of ΔV and aeolian transport potential are weak compared to the correlations with storminess. Also, except for a few profiles on wide beaches, the correlations are dominantly negative whereas positive correlations would be expected considering the positive dependence of aeolian transport on wind velocity. The negative correlations can be explained by the high impact of storm winds associated with storm surges. As transport potential is related to the cube of shear velocity, strong winds contribute exponentially to the yearly sum of potential transport. However, although these winds are theoretically capable of transporting large volumes of sediment, they also generate high sea levels and wave run up, reducing fetch distance and increasing surface moisture. Hence, actual aeolian transport is reduced [16]. Recalculations of transport potential with an upper limit on wind velocity decreased the proportion of negative correlations, in support of this proposition. Best results were obtained when years with high water levels (higher likelihood of dune erosion) were discarded. Hence, when the influence of dune erosion is low, aeolian transport potential can explain some of the year-to-year variability in dune-volume changes.

Further work as done by Delgado-Fernandez and Davidson-Arnott (2011) [16] is needed to identify aeolian transport activity in relation to wind velocity and sea levels at a timescale of days to months. Such higher-resolution records will enable better distinction between storm and fair-weather circumstances, recognition of the influence of bar-welding, and identification of spring versus neap conditions. On the basis of hourly values of wind velocity and sea levels, aeolian transport events can be discriminated from non-transport events, leading to better predictions of meso-scale sediment input to the dunes.

### The Effect of Local Beach Width

On a scale of decades, beach sections with W<200 m show positive correlations between W and ΔV. Other studies on beach and dune dynamics show similar results. In a study on dune dynamics on the Holland Coast (beach widths of 80–90 m), De Vries et al. (2012) [6] found positive correlations between beach slope and ΔV and suggest this is related to the limiting effect of beach slope on aeolian transport. For a sand spit in Lake Eerie (beach widths <40–90 m), Davidson-Arnott and Stewart (1987) [25] found that sand waves associated with bar welding offered both better protection against dune erosion and larger sediment input to the foredunes.

However, the results presented here indicate that when dune accretion dominates (i.e. low S), ΔV is constant across beach widths. In erosion-dominated years (high S), on the other hand, the relation between W and ΔV is especially evident. This implies that beach width or associated factors influence the extent of dune erosion and that the correlation between ΔV and W represents the effect of W on dune erosion instead of any effects on dune accretion.

The absence of a correlation between W and ΔV in calm years indicates that there is little impact of W on sediment supply to the dunes. This suggests that the critical fetch distance is considerably shorter than the minimum beach widths in the study area, or that even on relatively narrow beaches, there is sufficient sediment supply for aeolian transport to the dunes [61]. However, where fetch is a limiting factor, accretion may exhibit a stronger dependence on W than was observed here.

Where W>200 m (i.e. Terschelling and Schiermonnikoog), the influence of dune erosion is limited. The observed negative correlations between W and ΔV for these sites are unexpected. A possible explanation is the influence of transport-inhibiting topography on aeolian transport. Where beaches are very wide (W>300 m), shoreline-parallel depressions can be moist to wet, featuring persistent surface water after high tides. This inhibits aeolian transport, similar to observations of transport limitation on ridge-and-runnel beaches [62]. Furthermore, groundwater drainage during low tide on wide, low sloping beaches can lead to extensive moist zones, limiting aeolian transport even on the upper beach [32].

**Figure 12:**
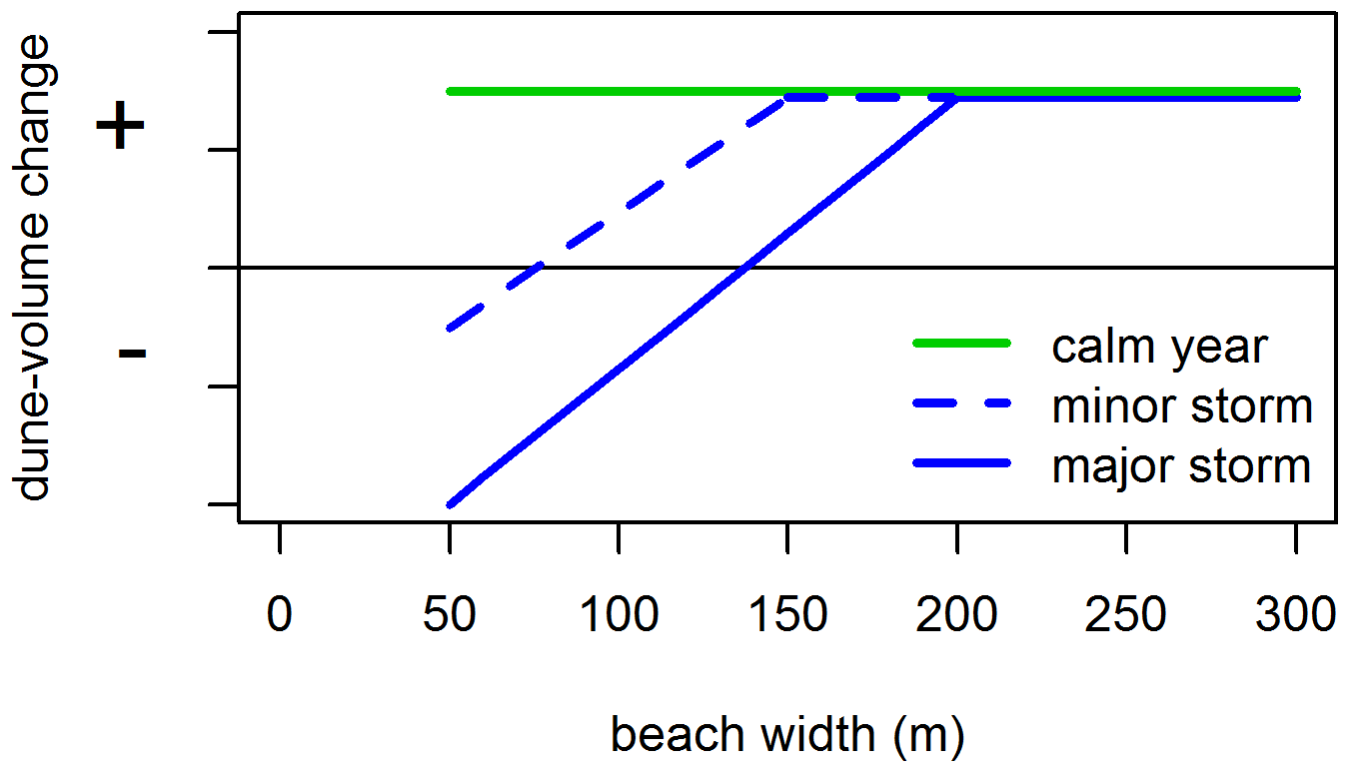
Schematic illustration of the influence of storminess and beach width on foredune volume. When beach widths are less than 200ΔV depends on storminess. When beach width is over 200 m, as occurs e.g. on Terschelling and Schiermonnikoog, ΔV is no longer dependent on storminess or beach width.

Bringing together the results presented in this paper, effects of regional climate (storminess) and local topography (beach width) can be integrated and summarised graphically, linking spatio-temporal variability in ΔV to variations in S and W (Fig. 12). This diagram synthesises observations from all sites and years, going from narrow to wider beaches and calm to stormy years. First, ΔV was found to be positive and relatively constant across all W in calm years (low S, green line). In stormy years (high S), ΔV is negative at narrow beaches and increases with W (blue lines). Foredunes backing beaches wider than 200 m (e.g. Terschelling, Schiermonnikoog) rarely experience erosion and ΔV is therefore positive, irrespective of S.

## Conclusions

Using a dataset of yearly beach-dune elevation profiles, temporal and spatial variability in dune-volume changes (ΔV) were calculated for six sections along the Dutch coast. Comparison of monitoring records shows that:

- Where beach width (W) is less than 200 m, temporal variability in ΔV is significantly correlated with yearly maximum sea levels; a proxy for storminess. Correlations with transport potential are weak at best.
- Beach width (W) is positively related to ΔV in years dominated by dune erosion. Hence, the impact of dune erosion is stronger on narrow beaches. Such a correlation is absent in years dominated by dune accretion, suggesting equal rates of aeolian sediment transport across all beach widths.
- Where fetch is not a limiting factor, alongshore variability in dune-volume changes is related more to dune erosion than dune accretion.
